# Supplementary material for: Heterogeneity of Phase II Enzyme Ligands on Controlling the Progression of Human Gastric Cancer Organoids as Stem Cell Therapy Model
Source: Int J Mol Sci. 2023 Nov 2;24(21):15911. doi: 10.3390/ijms242115911 (PMC10647227; doi:10.3390/ijms242115911)
Supplement: Supplementary file 1 [file ijms-24-15911-s001.zip › ijms-2650076-supplementary.pdf]

Supplemental Information

Heterogeneity of phase II enzyme ligands on controlling the progression of human gastric cancer organoids as stem cell therapy model

Deng-Chyang Wu[1-4], Chia-Chen Ku[1-3], Jia-Bin Pan[1-3], Kenly Wuputra[1-3], Ya-Han Yang[2,4], Chung-Jung Liu[2-5], Yi-Chang Liu[3], Kohsuke Kato[6], Shigeo Saito[7], Ying-Chu Lin[8], Inn-Wen Chong[1,9,10], Michael Hsiao[11], Huang-Ming Hu[4,12], Chao-Hung Kuo[4,13], Kung-Kai Kuo[2,4], Chang-Shen Lin[1], and Kazunari K. Yokoyama[1-3]*

[1]Graduate Institute of Medicine, [2]Regenerative Medicine and Cell Therapy Research Center, Kaohsiung Medical University, Kaohsiung 807, Taiwan, [3]Cell Therapy and Research Center, Kaohsiung Medical University Hospital, Kaohsiung 807, Taiwan, [4]Division of General and Digestive Surgery, department of Surgery, Kaohsiung Medical University Hospital, Kaohsiung 807, Taiwan, [5]Division of Gastroenterology, Department of Internal Medicine, Kaohsiung Medical University Hospital, Kaohsiung 807, Taiwan. [6]Department of Infection Biology, Graduate School of Comprehensive Human Sciences, the University of Tsukuba, Tsukuba 305-8577, Japan. [7]Saito laboratory of Cell Technology, Yaita 239-1571, Tochigi, Japan, [8]School of Dentistry, Kaohsiung Medical University, Kaohsiung 807, Taiwan,[9]Division of Pulmonary and Critical Care Medicine, Kaohsiung Medical University Hospital, Kaohsiung 807, Taiwan, [10]Department of Biological Science and Technology, National Yang Ming Chiao Tung University, Hsinchu 300193, Taiwan. [11]Genome Research Center, Academia Sinica, Nangan, Taipei 115, Taiwan, [12]Department of Internal Medicine, Kaohsiung Municipal Ta-Tung Hospital, Kaohsiung 80145, Taiwan, [13]Department of Internal Medicine, Kaohsiung Municipal Siaogang Hospital 812, Taiwan.

*Authors to whom correspondence should be addressed; Kazunari K. Yokoyama (kazu@kmu.edu.tw); Cell Therapy and Research Center, Kaohsiung Medical University Hospital; Department of Medicine, Graduate Institute of Medicine, Kaohsiung Medical University, Taiwan, Tel; +886-7312. 1001, ext 2729; FAX; +886-7313-3849). ORCID of C-C Ku; 0000-0002-1496-3081; C-S Lin; 0000-0001-7415-2187; K.K. Yokoyama; 0000-0001-8508-7587.

*Authors to whom correspondence should be addressed; Kazunari K. Yokoyama (kazu@kmu.edu.tw; Cell Therapy and Research Center, Kaohsiung Medical University Hospital; Graduate Institute of Medicine, Kaohsiung Medical University, Taiwan, Tel; +886-7312. 1001, ext 2729; FAX; +886-7313-3849). ORCID of C-C Ku; 0000-0002-1496-3081; C-S Lin; 0000-0001-7415-2187; K.K. Yokoyama; 0000-0001-8508-7587.

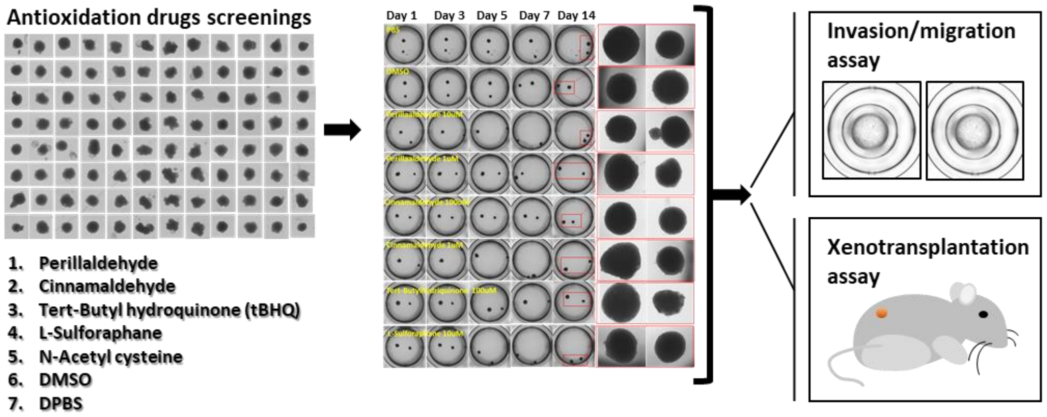

Supplemental Figure S1.

Imaging of drug screening of 3-D organoids cultivation. The ~500 μm of organoids which were generated by digestion with TrypLE reagents and seeded organoids sparsely in Transwells as monolayers (~30 organoid fragments per dome of ~20 μL, about 50,000 cells) with one dome per well of a 24-well plate. For the xenotransplantation assay, we injected organoids in scid mice (~100 organoid fragments per dome of ~200 μL, about 150,000 cells) [56].

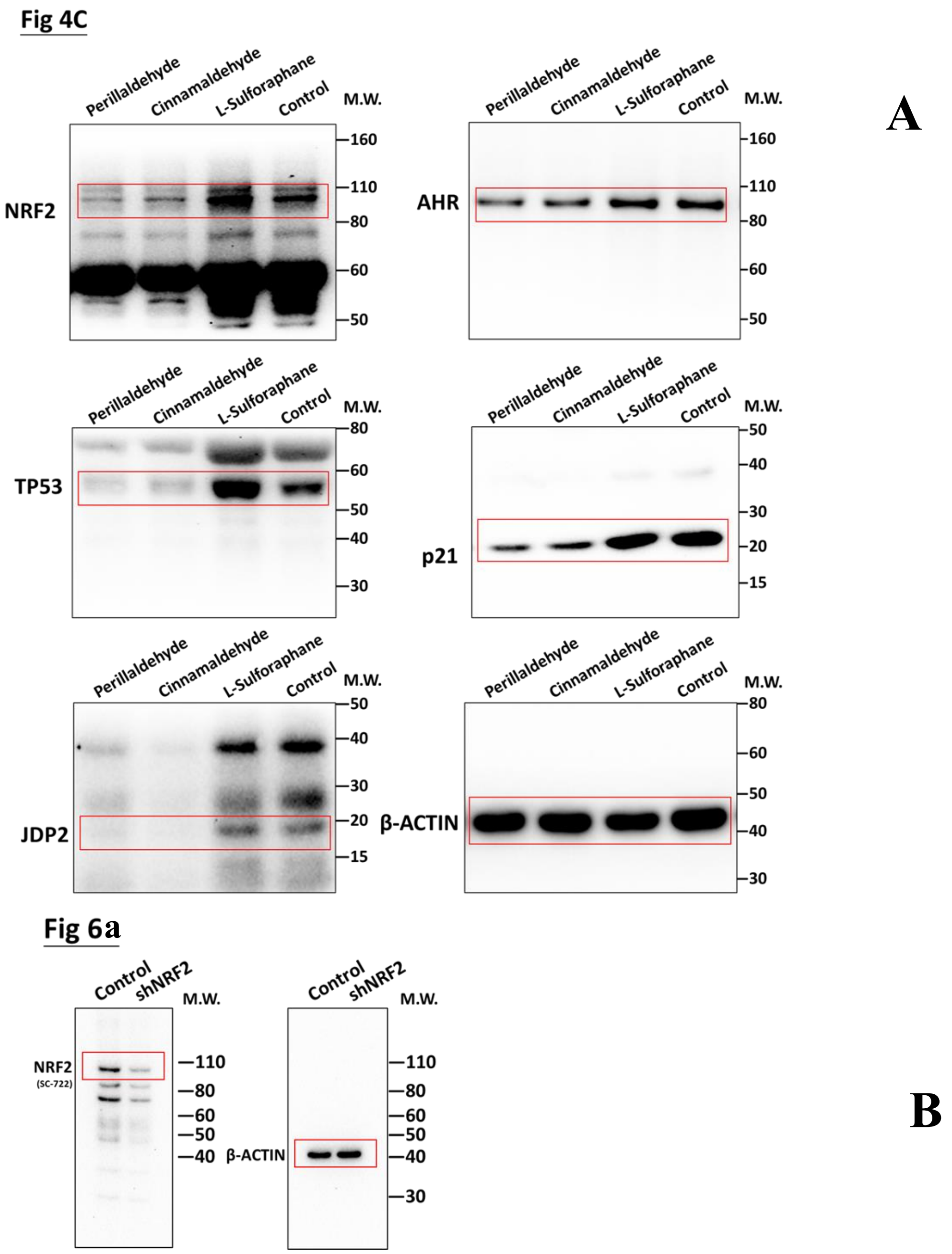

Supplemental Figure S2.

Uncropped raw data of western blots that were used in this article. Results are provided according to the sequence mentioned in the main text (**A**) Figure 4c and (**B**) Figure 6a. The red rectangles represent regions that were cropped to use in the figures of this article.
